# The success of using 2% lidocaine in pain removal during extraction of mandibular premolars: a prospective clinical study

**DOI:** 10.1186/s12903-020-01228-x

**Published:** 2020-08-31

**Authors:** Firas A. Jamil, Huda Moutaz Asmael, Mohammed Yahya Al-Jarsha

**Affiliations:** Department of Oral & Maxillofacial Surgery, Dental Teaching Hospital, College of Dentistry, University of Baghdad, Bab-Al Moadham, P.O.Box 1417, Baghdad, Iraq

**Keywords:** Buccal infiltration, Lidocaine, Articaine, Mandibular premolars, Inferior alveolar nerve block

## Abstract

**Background:**

The purpose of this study was to evaluate the anesthetic effectiveness of a buccal infiltration technique combined with local massage (using 2% lidocaine) in the extraction of mandibular premolars to be utilized as an alternative to the conventional inferior alveolar nerve block.

**Methods:**

Patients eligible included any subject with a clinical indication for tooth extraction of the mandibular 1st or 2nd premolars. All patients were anesthetized buccally by local infiltration technique followed by an external pressure applied for 1 min directly over the injection area. In each case, another local injection was given lingually. All operations were started at approximately 5 min after the buccal injection. The collected data included age, gender, pain perception and its intensity during treatment at three checkpoints, apical tenderness, and the type of extraction. Any associated complications or difficulties were also recorded. Then the results were analyzed and interpreted using appropriate statistical tests. The significance level was set at *P* ≤ 0.05.

**Results:**

A total of 247 cases (1st premolar, *n* = 119; 2nd premolar, *n* = 128), predominantly male, were included. In 95% of study sample, the patients were satisfied with the dental extraction without any pain. However, in 5% of cases, pain was reported at the stage of tooth removal. Apical tenderness was found to be present in 11% of the total cases. Three teeth required surgical removal. Upon analysis, no significant differences in the success rates were detected between the 2 premolar groups or amongst the various age groups. Minor and transient side effects were reported in this study.

**Conclusion:**

The technique is simple and effective as well. It might be considered as an alternative anesthetic injection to the inferior alveolar nerve block for dental extraction of the mandibular premolars.

## Background

Local anesthetic agents rank among the most frequently used drugs both in medicine and modern dentistry due to their pivotal role [1]. It is well known that a patients’ anticipation of pain may compromise dental treatment [2] and that the action of local anesthetic administration typically triggers anxiety [3].

The inferior alveolar nerve block (IANB) is the most widely used anesthetic technique in the posterior mandible [4]. It provides profound anesthesia to perform surgical and restorative procedures in that area when it is administered successfully [5]. The reported success rates for IANB in the literature varies widely due to multiple factors including the type of anesthetic agent, operator experience and familiarity with the technique, anatomical variations, patient’s anxiety level, teeth pulp status, study sample size, and the criteria for identifying the successful outcome [6–8]. Many modifications have therefore been implemented to increase these success rates and make them more predictable. Such modifications include changing the type of anesthetic solution [9, 10], its injected volume [11], composition e.g. buffering / twin mixing [12–14], combination with other supplemental anesthetic techniques [15, 16], changing patients’ position during injection [17], preceding the injection with oral premedication e.g. ibuprofen / ketorolac / meloxicam / tramadol / dexamethasone [11, 18–24], and/or following it with cryotherapy application [25]. However, conventional IANB is still associated with a relatively high reported failure rates ranging from 20 to 47% in the premolar area [17, 26]. Additionally, major intra- and postoperative complications such as systemic toxicity due to iatrogenic intravascular injections, severe bleeding as a result of adjacent blood vessels injury, prolonged mandibular anesthesia, as well as transient, or even permanent paresthesia of the inferior alveolar and the lingual nerves were recorded to be associated with this technique [27–29].

To avoid the abovementioned disadvantages, clinicians have investigated alternative anesthetic techniques such as periodontal intraligamentary injection (PDL) [2, 4]. It was found that PDL could be considered as a sufficient alternative to IANB for single tooth anesthesia providing a less painful method of injection without the risk for nerve damage [4] with a circumscribed effect on the adjacent soft tissue only [30]. However, an ideal PDL technique commonly requires specialized high-pressure syringe systems or computer-controlled local anesthetic devices with special 30-gauge short needles [2, 4, 30]. In addition, damages to the periodontal tissue and root resorption [31] as well as severe bacteremia up to 100% were reported [32]. Another study for initial anesthetic administration via buccal infiltration in the posterior mandible with a pressure syringe (P-INF) has showed a success rate of only 35% with a lower anesthetic efficacy and significant necessity for second injections compared to IANB [33].

It is essential that local anesthetic agent provides rapid onset, single tooth anesthesia (i.e. be limited in its effect to the site needed), safety, efficiency with low toxicity, as well as minimal complications. Therefore, the aim of this study was to evaluate the anesthetic effectiveness and simplicity of a buccal infiltration technique, using 2% lidocaine as the sole agent, in the extraction of the mandibular premolars in specific as a potential alternative to the IANB technique for minimizing the complications that are usually associated with the latter procedure.

### Question in focus

The current study aimed to answer the following question: can a buccal infiltration with 2% lidocaine (combined with a lingual injection) replace the inferior alveolar nerve block in the extraction of mandibular premolars?

## Methods

### Study design

**Fig. 1:**
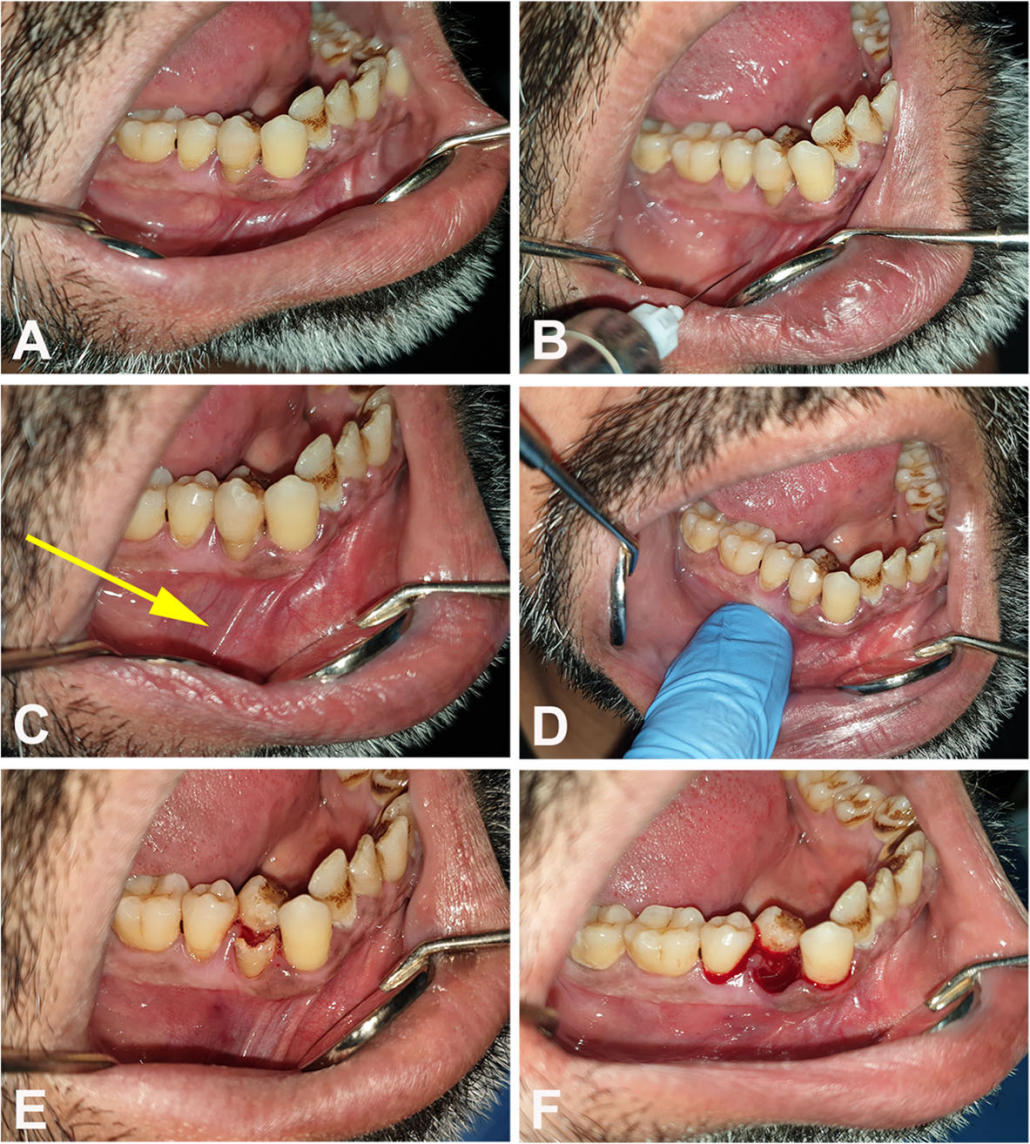
a Tooth no. 44 presented with severe pain and fractured crown due to extensive carious lesion; b needle insertion with injection of the anesthetic agent in the buccal vestibule; c the injection area directly after deposition of local anesthesia (*yellow arrow*); **d** simple pressure applied over the injection site using the operator’s index finger; **e** crown removal; and **f** extraction of the accused tooth

A non-comparative prospective case series study was conducted at the Department of Oral and Maxillofacial Surgery of the Dental Teaching Hospital at the College of Dentistry, University of Baghdad. The current study described the received intervention and outcomes for each patient separately; there was no control group as all subjects were enrolled in the study group to assess the efficiency of the anesthetic technique. The study protocol was formatted and approved at first, then patient recruitment started according to specific inclusion and exclusion criteria from April 2019 to March 2020. The inclusion criteria for patients were any case with clinical indication for tooth extraction in the mandibular 1st & 2nd premolars (Fig. 1a). Patients with allergy for local anesthetic components, 2 adjacent premolars that are both indicated for extraction, physical or mental disabilities, limitation in mouth opening, chronic abuse of any medication that could affect the pain threshold, as well as swelling in and around the area of injection were excluded from this study. A verbal questionnaire and a written medical history were obtained to ensure the fulfillment of these criteria followed by a detailed clinical examination conducted by the operating author. Prior to the commencement of the procedure, all patients were informed about the possibility of unpleasant pain sensation during the surgical procedure and then all willing patients had signed a written informed consent for participation in this study. Regarding younger patients (under 18 years-old), a written consent was also obtained from their guardians. All procedures were conducted by a single well-experienced surgeon. Each participant received dental treatment for one tooth only. The ethical approval for this study was obtained from the Research Ethics Committee (Reference no.: 090119) at the College of Dentistry, University of Baghdad.

### Anesthetic technique

The local anesthetic agent (2.2 mL cartridges of lidocaine hydrochloride 2% with adrenaline 1:100,000, Septodont, France) was administered to all patients in the same manner; in the buccal vestibule against the apex of the accused premolar inserting the needle until it touches bone (Fig. 1b). Then, 1/2 cartridge (about 1.1 mL) of the anesthetic solution was deposited slowly by local infiltration technique followed by a pressure application for 1 min (using the operator’s index) over the alveolar gingiva (Fig. 1d). Another local injection (1/4 cartridge, about 0.5 mL) was given lingually immediately following the first buccal injection. All interventional procedures started at approximately 5 min, after the buccal injection. A PDL and/or intrapulpal injection were only done (as supportive injections) for failed cases in which pain sensation was elicited during treatment. In such cases, if the pulp chamber was already accessible then intrapulpal injection was administered, otherwise, PDL injection was selected as the supportive injection choice. If in one of the cases all the techniques above had failed to provide adequate anesthesia, it was planned that an IANB injection would be administered to complete the extraction procedure.

### Data collection and statistical analysis

The parameters recorded in this study included the demographic data (age, gender), the presence or absence of tenderness to apical percussion prior to treatment, pain perception during the procedure at 3 time points (gum piercing while checking for the adequate soft tissue anesthesia, gingival separation, and tooth removal), pain intensity using a visual analog scale (VAS) with an upper limit of 10, anxiety for two times injections, the need for second supportive injection, and the type of the completed extraction procedure (normal/surgical). The presence of any associated complications or difficulties were also recorded for each case. All patients were asked to contact the clinic as soon as the numbness wears off. Patients with minor complications were followed up for few days after the procedure to determine the need for additional interventions if necessary.

For the purpose of statistical analysis, all cases included in this study were divided into 2 groups (1st premolar and 2nd premolar groups) to evaluate the effectiveness of this technique in relation to the tooth extraction location. The 1st premolar and the 2nd premolar groups were further categorized into 6 age groups to detect any effect of the patient’s age on the success rate with this technique.

The statistical analysis was performed using GraphPad Prism Software version 8.0 (GraphPad Software Inc.). Descriptive statistics of range, frequency, mean ± standard deviation (SD) were calculated for the study sample. Fisher’s test was applied to detect any statistical differences in the success rate between the 2 main groups as a whole. Chi-square test was applied to detect any statistical differences in the success rate between the 6 age categories within each main group. All probability levels were considered significant at *P* value ≤0.05.

## Results

**Fig. 2:**
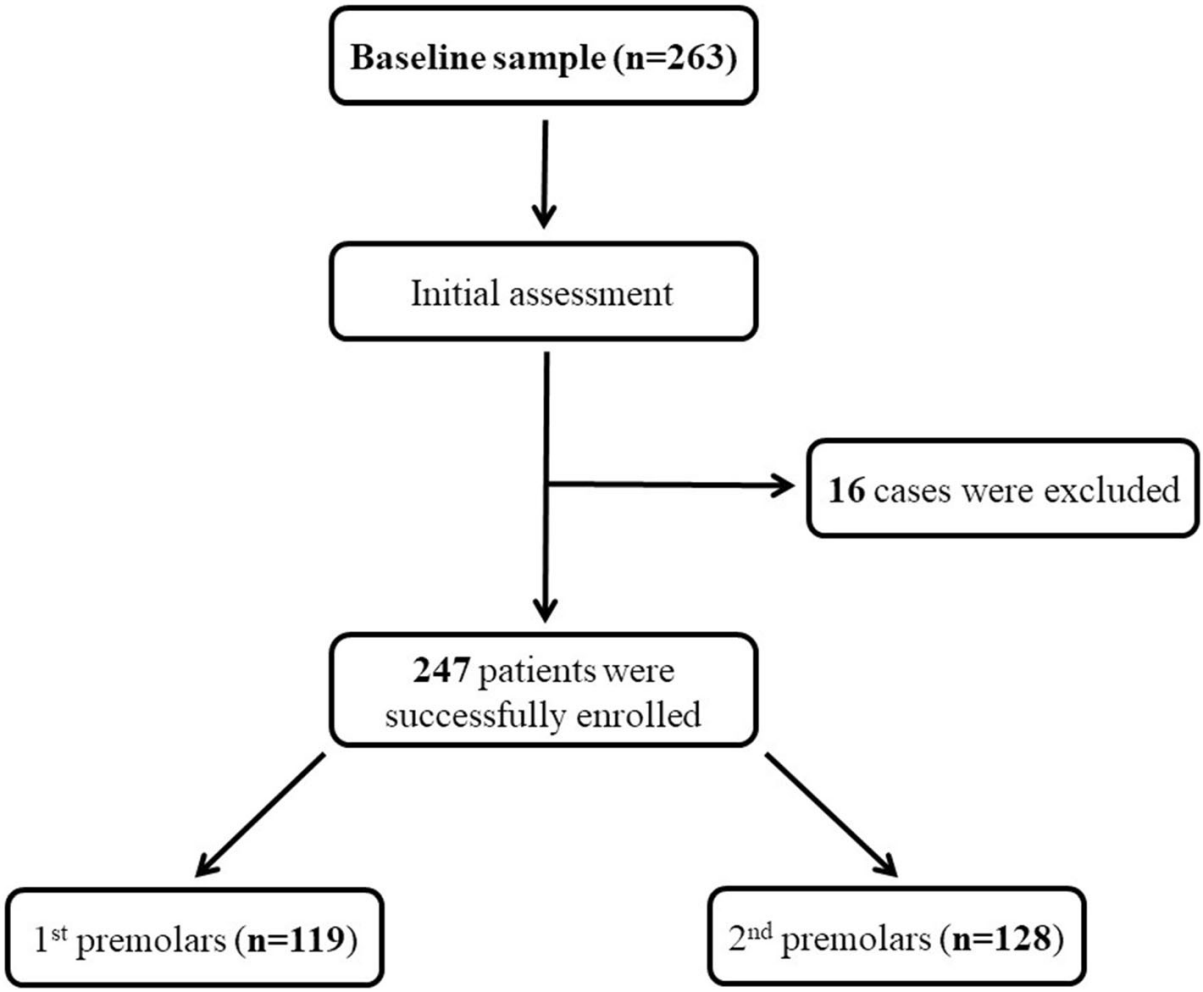
Flow chart briefly illustrated the study sample and patients enrollment

The total number of subjects enrolled in this study was 247 (141 male, 106 female; age range, 15 to 75 yr; mean age, 43.88 ± 14.02 yr). A detailed flow chart representing the sample size and patients enrollment is illustrated in Fig. 2. Other descriptive statistics are presented in Table 1. Only three cases were removed surgically (transalveolar extraction) while the other 244 cases were treated by normal (intraalveolar) extraction.

**Table 1:** Descriptive statistics of the study variables with frequencies of apical tenderness and pain perception

Cases in total n (%)	Gender n (%)		Mandibular premolars n (%)		Pain n (%)		Apical tenderness in total n (%)
247 (100)	Male	Female	1st	2nd	with	without	26 (11)
	141 (57)	106 (43)	119 (48)	128 (52)	13 (5)	234 (95)	

*n* number of occurrence

Table 2 shows the results of pain perception recorded at the three steps of treatment (gingival piercing, separation of soft tissues, tooth removal) together with the supportive injections needed in the failed cases of both groups. In the 1st premolar group, 4 patients reported pain during tooth removal step (VAS ranged 2 to 7), 3 of which had tenderness to apical percussion prior to treatment. In the 2nd premolar group, 9 patients reported pain at the same step (VAS ranged 1 to 6), 7 of which had tenderness to apical percussion preceding treatment. Three cases in total had to undergo a surgical extraction due to ankylosis; their removal were completed without the need for any supportive injections.

**Table 2:** Pain perception during dental extraction procedure in association with the supportive injections

Group	Pain n (%)			No pain n (%)	Supportive injection n (%)		
	Gingival piercing	Soft tissue separation	Tooth removal		PDL	Intrapulpal	IANB
1st Premolar	0 (0)	0 (0)	4 (3)	115 (97)	3 (3)	1 (1)	0 (0)
2nd Premolar	0 (0)	0 (0)	9 (7)	119 (93)	6 (5)	3 (2)	0 (0)

*n* number of occurrence

*PDL* periodontal intraligamentary

*IANB* inferior alveolar nerve block

The results showed a statistically non-significant differences in the success rates (extractions without pain) between the 2 premolar groups (*P* = 0.2582). In addition, there was no important difference in the success rate amongst the various age groups (Table 3).

**Table 3:** Comparison of the success rates amongst different age groups

Age Groups yr (n)	Successful cases of 1st premolar	Successful cases of 2nd premolar	Total success rate n (% within group)	(*P* value)
15–24 (29)	15	13	28 (97)	0.3650^a,†^
25–34 (39)	22	12	34 (87)	
35–44 (56)	24	30	54 (96)	
45–54 (54)	23	31	54 (100)	
55–64 (45)	18	22	40 (89)	
65–75 (24)	13	11	24 (100)	

*n* number of occurrence

^a^ By Chi-square

^†^ Non-significant

Hematoma (a small vesicle of blood under the gingiva at the site of injection) ranged 2 to 5 mm was observed in 7 cases in total. In addition, paresthesia (temporary numbness in lower lip) ranged about 1.8 to 3.4 h (mean time = 2.3 h) was also reported. Upon follow up, both hematoma and paresthesia were resolved spontaneously without any further intervention. None of the patients reported any additional anxiety due to the need for a second anesthetic injection. Furthermore, there was no any toxic reaction or adverse effect of the local anesthetic drug used in this study.

## Discussion

In light of the variable and high reported failure rates of IANB technique and its many possible complications [28, 34–41], alternative techniques were investigated in the literature [2, 4, 42]. However, most of these alternatives were complex and/or demanding special devices [2, 4, 30], and had their own complications [31–33]. Therefore, another technique with adequate anesthetic efficiency and low complexity is needed. Despite some studies have evaluated similar techniques (buccal infiltration) as an alternative to IANB in anesthetizing the mandibular posterior teeth [33, 43–50], most of them either used 4% articaine alone [33, 43, 44, 47, 50], or preferred it over lidocaine as the anesthetic agent [46, 48, 49]. In dentistry, 4% articaine has been used as a local anesthetic agent for many years and it was reported to be better in ensuring pulpal anesthesia than a 2% lidocaine solution. It can penetrate the cortical bone due to its unique chemical structure (thiophene group) which increases its liposolubility [51, 52]. Therefore, it was considered as a good alternative to lidocaine for healthy teeth and for patients with symptomatic pulpitis [48]. However, articaine still had its downside of prolonged soft tissue anesthesia (paresthesia) reported after the conclusion of the procedure [5, 53], making them more susceptible to self-injury in addition to be more annoying to the patient to the point that requires ansethetic reversal in some situations [54–56]. Besides, lidocaine has shown to have a wider margin of safety than articaine according to a recent meta-analysis study [10].

The present study has assessed the anesthetic efficiency of buccal infiltration technique (combined with lingual regional anesthesia) using 2% lidocaine with epinephrine (1:100,000) as the sole agent for extraction of mandibular premolars. In the literature, all the previous studies that utilized lidocaine for the same purpose have reported much lower success rates than the one under study. The efficiency of lidocaine diffusion in our procedure could be attributed to the buccally applied pressure by the operator’s index over the injection site. This maneuver may enforce a larger amount of the local anesthetic agent to be in contact with a wider surface area of the bony cortical plate for an extra length of time. In consequence, larger concentrations of the anesthetic drug would be expected to provide an effective hard tissue anesthesia rather than losing a considerable amount of the solution into the surrounding soft tissue as with the conventional infiltration techniques. However, further investigations are needed to explain the success results with our proposed technique as it is well established that the cortical bone in this area is relatively dense [49, 57].

The authors had conducted the study technique for a large sample size to have the proper power of statistically detecting small differences. Interestingly, pain perception (as an indication of technique failure) was not reported by most of the patients (95%, Table 1), specifying that this technique would be a promising procedure for extracting the mandibular premolars.

In our study sample, there were no recorded cases of pain perception during gingival piercing or soft tissue separation (Table 2). However, failed initial anesthesia has been detected at the tooth removal stage (3% of 1st premolars, and 7% of 2nd premolars groups). Most of these cases (10 out of 13) had been tender to apical percussion prior to anesthetic agent administration. The failed cases were managed successfully by an additional technique (PDL/intrapulpal) to complete the extraction procedure (Table 2). These simple supportive procedures seemed to be very effective especially for cases that are associated with periapical pathology, eliminating the need for IANB. These findings contrast those of Aggarwal et al. [45] who suggested the use of a combination of buccal infiltration and IANB to overcome the anesthetic failure in symptomatic mandibular premolars.

Another interesting observation is that IANB was not needed at all even with surgical extractions. This could be explained by the fact that the required surgical extraction procedure for these teeth is relatively short [49] and simple (i.e. bone removal buccally after flap reflection to create a point of application for a dental elevator) in addition to its being involving buccal tissues only, the same area where the first injection had been originally deposited.

No statistical significant difference (*P* > 0.05) in the success rates was found between the two premolar groups (97% for 1st premolar, and 93% for 2nd premolar). These results discourage the use of a different anesthetic technique for each tooth. It was well-established that mandibular cortical bone thickness as well as its trabecular density increase with age [58, 59]. This observation could affect local anesthetic penetration and effectiveness when used with a buccal infiltration technique in the mandible [60]. To address this issue, the success rates amongst different age categories were compared. Although they were slightly lower in the 25–34 year (87%) and the 55–64 year age categories (89%), the statistical analysis showed non-significant differences as compared to the other age groups (Table 3). Again, supporting that this technique could be suitable for any patient age.

All possible anesthetic complications were observed and registered when present to properly assess any side effects arising from the application of the study technique. It is worth mentioning that no major side effects were reported in our study except for a few cases of small sub-gingival hematomas (3%) which resolved spontaneously without the need for any further interventions. The mean duration of lower lip numbness (2.3 h) was shorter to that reported by Kämmerer et al. [5] (3.8 h) who recorded a significant longer duration of soft tissue anesthesia in the cases that anesthetized by 4% articaine with epinephrine (1:100,000). As opposed to the mental-incisive nerve block, no effort was made in our technique to insert the needle’s tip into the mental foramen. Thus, avoiding the possibility of iatrogenic damage to the mental nerve and blood vessels. In addition, it was well tolerated by patients as it avoided the unpleasant burning pain of giving the local anesthetic injection directly into the mental foramen.

## Conclusions

To conclude, this study showed that buccal infiltration technique (combined with simple index finger pressure) using lidocaine plus lingual anesthesia, might be a good alternative to the IANB in anesthetizing mandibular premolar teeth prior to their extraction. The proposed technique does not rely on the assistance of any premedication. The simplicity of this maneuver encourages its use in the dental extraction of mandibular premolars, particularly for dental students and juniors (i.e. it provides easy and short learning curve).

However, within the limitations of this research, further studies with a larger sample size and a control comparison group (using IANB) from the same study population are recommended.

## Data Availability

The datasets used and/or analyzed during the current study are available from the corresponding author on reasonable request.

## Abbreviations

IANB: Inferior alveolar nerve block
PDL: Periodontal intraligamentary injection
VAS: Visual analog scale
SD: Standard deviation
